# A mediator-free sonogenetic switch for therapeutic protein expression in mammalian cells

**DOI:** 10.1093/nar/gkaf191

**Published:** 2025-03-20

**Authors:** Jinbo Huang, Shuai Xue, Ana Palma Teixeira, Martin Fussenegger

**Affiliations:** Department of Biosystems Science and Engineering, ETH Zurich, Klingelbergstrasse 48, CH-4056 Basel, Switzerland; Department of Biosystems Science and Engineering, ETH Zurich, Klingelbergstrasse 48, CH-4056 Basel, Switzerland; Department of Biosystems Science and Engineering, ETH Zurich, Klingelbergstrasse 48, CH-4056 Basel, Switzerland; Department of Biosystems Science and Engineering, ETH Zurich, Klingelbergstrasse 48, CH-4056 Basel, Switzerland; Faculty of Science, University of Basel, Klingelbergstrasse 48, CH-4056 Basel, Switzerland

## Abstract

An ultrasound-responsive transgene circuit can provide non-invasive, spatiotemporally precise remote control of gene expression and cellular behavior in synthetic biology applications. However, current ultrasound-based systems often rely on nanoparticles or harness ultrasound's thermal effects, posing risks of tissue damage and cellular stress that limit their therapeutic potential. Here, we present Spatiotemporal Ultrasound-induced Protein Expression Regulator (SUPER), a novel gene switch enabling mediator-free, non-invasive and direct regulation of protein expression via ultrasound in mammalian cells. SUPER leverages the mammalian reactive oxygen species (ROS) sensing system, featuring KEAP1 (Kelch-like ECH-associated protein 1), NRF2 (nuclear factor erythroid 2-related factor 2), and antioxidant response element (ARE) as its core components. We demonstrate that low-intensity (1.5 W/cm^2^, ∼45 kHz), brief (40 s) ultrasound exposure generates non-toxic levels of ROS, activating the KEAP1/NRF2 pathway in engineered cells and leading to the controlled expression of target gene(s) via a synthetic ARE promoter. The system exhibits robust expression dynamics, excellent reversibility, and functionality in various cell types, including human mesenchymal stem cell-derived lines (hMSC-TERT). In a proof-of-concept study, ultrasound stimulation of subcutaneously implanted microencapsulated engineered cells stably expressing the sonogenetic circuit in a type 1 diabetic mouse model triggered sufficient insulin production to restore normoglycemia. Our work highlights ultrasound's potential as a precise and non-invasive tool for advancing cell and gene therapies in personalized medicine.

## Graphical Abstract

**Graphical Abstract:**
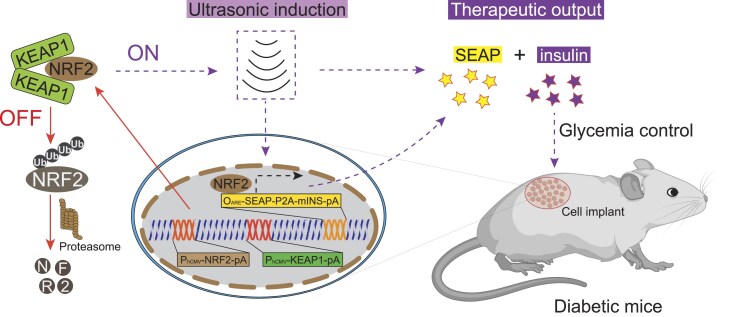

## Introduction

Synthetic biology has revolutionized cell-based gene therapy by enabling the design and construction of artificial gene circuits for therapeutic protein regulation and cellular behavior control in response to specific inputs [1–3]. Traditional inducible gene systems utilize a range of chemical inducers [4–6], such as doxycycline [7], abscisic acid (ABA) [8], and rapamycin [9], to achieve reliable and tunable gene regulation. However, these systems often depend on systemic administration of the inducer, raising concerns about tissue specificity and off-target effects, as well as presenting challenges in achieving sustained control [2, 10–12]. As alternatives to chemical cues, recent advances in synthetic biology have explored physical stimuli, including light [13, 14], heat [15, 16], magnetic fields [17, 18], mechanical vibration [19, 20], and electrical currents [21–24], as inducers of gene expression. These approaches, termed optogenetics [13, 14], thermogenetics [15, 16], magnetogenetics [17], mechanogenetics [19, 20], and electrogenetics [21–23], respectively, offer new avenues for precise and reversible gene regulation. However, each approach has characteristic limitations. For example, optogenetics is limited by the shallow tissue penetration of light and the requirement for invasive optical hardware [25, 26]. Electrogenetics and thermogenetics similarly offer precise temporal control, but carry risks of cell damage due to exposure to electrical or thermal stress [15, 16, 21, 27]. Magnetogenetics, while enabling remote activation, usually depends on nanoparticles for efficient gene modulation, which can pose safety concerns related to long-term biocompatibility [17, 28].

In this context, ultrasound offers several compelling advantages as a non-invasive, deeply tissue-penetrating and tuneable physical cue to regulate gene expression. Ultrasound is well established in biomedical applications as an imaging modality [29, 30], and has more recently been explored for regulation of gene expression [15, 31–33], cellular activities [34, 35], body temperature [36], and host behaviours [37]. However, current ultrasound-based gene regulation technologies generally rely on indirect mechanisms. Some methods utilize ultrasound's mechanical force or heating effect to trigger gene expression, but these approaches can risk unintended tissue damage or cellular stress [15, 31, 34, 35, 38]. Other systems, like sonodynamic therapy, depend on nanoparticle-mediated enhancement of reactive oxygen species (ROS) production, which complicates the approach by introducing delivery challenges and potential safety issues related to nanoparticle biocompatibility [39, 40]. These limitations underscore the need for a more straightforward, non-invasive, and safe ultrasound-based gene switch that can regulate gene expression precisely without the need for auxiliary agents or invasive procedures.

Most recent studies on ultrasound-induced ROS generation emphasize the use of sonosensitizing nanoparticles or specialized materials to enhance ROS production [39, 40], especially for biomedical applications. In contrast, we aimed to harness the intrinsic effects of ultrasound itself—specifically ultrasonic pressure, shear stress, acoustic streaming, and cavitation—to fine-tune ROS generation without external mediators [41–45]. These ultrasound-induced effects stimulate intracellular free radical formation, directly producing controlled and biosafe levels of ROS within cells [41–43]. Leveraging this ROS generation, we developed an ultrasound-regulated gene switch centered on an engineered ROS-sensing system, which consists of Kelch-like ECH-associated protein 1 (KEAP1), nuclear factor erythroid 2-related factor 2 (NRF2) and antioxidant response element (ARE)-controlled effectors [46, 47]. Upon low-intensity ultrasonic (1.5 W/cm²) stimulation, oxidative stress induces dissociation of the KEAP1-NRF2 complex, resulting in the release of NRF2 and its translocation to the nucleus, where it binds to the ARE to activate the expression of a protein of interest (P.O.I), enabling precise, mediator-free and non-invasive gene regulation in mammalian cells.

Our developed circuit, termed Spatiotemporal Ultrasound-induced Protein Expression Regulator (SUPER), was validated both *in vitro* in cell culture and *in vivo* in a type 1 diabetic (T1D) mouse model. The system exhibits robust expression dynamics, and applicability to various cell types, including a human mesenchymal stem cell-derived line (hMSC-TERT). In a proof-of-concept study, we subcutaneously implanted engineered monoclonal cells stably expressing the KEAP1/NRF2/ARE-responsive ROS system in a T1D mouse model. Upon ultrasonic induction, the SUPER system produced sufficient insulin to restore and maintain normoglycemia through the whole treatment period, highlighting its potential as a versatile, mediator-free, and non-invasive gene regulation tool for precision medicine.

## Materials and methods

**Construction of plasmids**. All expression vectors were designed using the Benchling platform (www.benchling.com). Plasmid information is provided in Supplementary Table S1. DNA fragments were amplified with Q5 High-Fidelity 2x Master Mix (M0492L, New England BioLabs). The amplified DNA was purified using a DNA recovery kit (D4002, Zymo Research). Restriction enzyme digestion of the vectors was performed with enzymes from NEB, followed by ligation of the backbones and inserts using Gibson Assembly Master Mix (E2611L, New England BioLabs). The ligation products were transformed into competent XL10-Gold K12 *Escherichia coli* cells (Stratagene). The cells were incubated on ice for 10 min, then heat-shocked at 42°C for 90 s. Transformed bacteria were plated on Luria-Bertani (LB) agar containing the appropriate antibiotics. Individual colonies were selected and grown in liquid LB culture, and plasmids were extracted using a plasmid purification kit (D4037, Zymo Research). The purified plasmids were commercially sequenced by Microsynth AG (Balgach, Switzerland).

**Cell culture and transfection**. In this study, all mammalian cells were cultured in Dulbecco's modified Eagle's medium (DMEM, cat. no. 52100–39, Thermo Fisher Scientific) supplemented with 10% fetal bovine serum (FBS, cat. no. F7524, lot no. 022M3395, Sigma-Aldrich) and 1% (v/v) streptomycin/penicillin (cat. no. L0022, Biowest) at 37°C in a humidified atmosphere containing 5% CO_2_. For transfection, 50 000 cells were seeded per well in a 24-well plate (cat. no. 3599, Corning Inc. Life Sciences) and allowed to grow for 24 h. Transfection was performed by adding 40 μL of a transfection mixture consisting of 2 μg polyethyleneimine (PEI MAX®, MW 40 000, stock solution 1 μg/μL in ddH_2_O, cat. no. 24765–2, Polysciences) and 0.5 μg plasmid DNA (or equimolar concentrations for plasmid mixtures). After 8 h, the transfection medium was replaced with 500 μL of fresh culture medium. The following cell lines were used in this study: human embryonic kidney cells (HEK-293T, ATCC: CRL-11268), human telomerase-immortalized mesenchymal stem cells (hMSC-TERT), human fibrosarcoma cells (HT-1080, ATCC: CCL-121), human cervical adenocarcinoma cells (HeLa, ATCC: CCL-2), Chinese hamster ovary cells (CHO-K1, ATCC: CCL-61), and baby hamster kidney cells (BHK-21, ATCC: CCL-10).

**SEAP quantification *in vitro***. SEAP levels in the cell culture supernatant were quantified using a substrate-based colorimetric assay. Briefly, 100 μL of 2x SEAP assay buffer (composed of 20 mM homoarginine, 1 mM MgCl_2_, and 21% diethanolamine, pH 9.8) and 20 μL of substrate solution (120 mM p-nitrophenyl phosphate, cat. no. AC128860100, Thermo Fisher Scientific) were added to 80 μL of heat-inactivated (30 min, 65°C) cell culture supernatant. The mixture was immediately monitored for absorbance at 405 nm over a 30-min time course at 37°C using a Tecan Infinite 200 PRO plate reader. SEAP levels were calculated according to standard protocols.

**SEAP quantification *in vivo***. SEAP levels in the bloodstream were quantified using a SEAP reporter gene assay kit (cat. no. ab133077, Abcam, UK), following the manufacturer's protocol.

**ROS quantification**. Sonoinduced cells were first washed with 500 μL of phosphate-buffered saline (PBS, cat. no. 14190–094, Thermo Fisher Scientific). The cells were then incubated with 500 μL of FBS-free DMEM containing 25 μmol/L 2′,7′-dichlorofluorescein diacetate (cat. no. D6883, Sigma-Aldrich) for 45 min at 37°C. After incubation, the cells were washed again with 500 μL of PBS, and ROS levels were quantified by means of fluorescence assay (excitation/emission: 485/535 nm) on a plate reader (Tecan Infinite 200 PRO, Tecan Group AG).

**Cell viability**. Cell viability was assessed by incubating the cells with 50 μg/mL resazurin (cat. no. R7017, Sigma-Aldrich) for 1–2 h at 37°C. Fluorescence was measured at 540/590 nm using a plate reader (Tecan Infinite 200 PRO, Tecan Group AG).

**Protein expression kinetics**. The kinetics of SEAP reporter or therapeutic protein expression were assessed in engineered mammalian cells transfected with the ROS-sensing system. Following ultrasonic induction (1.5 W/cm^2^, ∼45 kHz) at the 0-h time point, samples were collected from the culture supernatant at specified intervals over 2 days (for stable cells) or 3 days (for transiently transfected cells) for SEAP reporter or therapeutic protein quantification. A single ultrasonic induction was applied exclusively at the 0-h time point.

**Reversibility evaluation of expressed protein**. The reversibility of protein expression in the ultrasound-induced system was evaluated by means of repeated ON/OFF treatment cycles. During each cycle, engineered mammalian cells were initially induced with ultrasound (sonoinduction, ON; 1.5 W/cm^2^, ∼45 kHz, 40 s), and samples were collected from the culture supernatant after 12 and 24 h for SEAP reporter or therapeutic protein quantification. After sample collection at 24 h, the culture medium was replaced with fresh medium, the cell density was adjusted to match the initial density, and the cells were cultured without ultrasound treatment (OFF) for the next 24 h. This procedure was repeated for subsequent induction cycles. Controls were treated similarly, but with no ultrasonic induction (OFF throughout).

**Monoclonal cell line generation**. In the initial phase, 5 × 10^4^ HEK293 cells were seeded in a 24-well plate and cultured for 24 h. The cells were then co-transfected with pJH1101 (200 ng), pJH1054 (550 ng), pJH1169 (400 ng), and pJH42 (P_hCMV_-SB100X-pA), which encodes constitutive expression of the SB transposase (200 ng). After 24 h, the cell culture medium was replaced with fresh medium containing puromycin (1 μg/mL), blasticidin (10 μg/mL), and zeocin (100 μg/mL) for a 3-day antibiotic selection process. Following antibiotic selection, the polyclonal population of genetically engineered cells was suspended in DMEM and subjected to fluorescence-activated cell sorting using a BD Biosciences system. After 2 weeks of expansion, the criterion of ROS-based activation was used to select the best-in-class cell lines for subsequent experiments.

**Ultrasonic induction *in vitro***. Cells were seeded in a 24-well plate and cultured for 24 h prior to transfection. After transfection, the cells were trypsinized and collected in a 15 mL Falcon tube (cat. no. E24043GH, Greiner Bio-one) containing 1 mL of complete medium. The Falcon tube with the engineered cells was then placed in the water bath of the sonicator system (cat. no. UCD-600, Diagenode, Belgium), which operates in the frequency range of 30–60 kHz with an output power range of 100–350 W (1.25–4.5 W/cm^2^). During ultrasonic induction, the water bath was maintained at 15–25°C with the use of ultrasound gel (cat. no. G00664, Tinovamed GmbH, Switzerland). The sonication program was set to 5 s on, 5 s off, with a total induction time of 5 s per cycle. Following ultrasonic induction, the cells were reseeded in 24-well plates for further culturing. Reporters in the cell culture supernatant were quantified 24 h post-sonication or at a specified time point.

**Real-time temperature monitoring**. During ultrasonic induction both *in vitro* and *in vivo*, the temperature of the Falcon tubes containing the engineered cells and the implantation site of the mice was continuously monitored using a digital infrared thermometer (cat. no. 206–8741, RS PRO, UK).

**Quantitative PCR (qPCR)**. Approximately 1 million cells were cultured overnight in a six-well plate. The following day, the cells were trypsinized and collected into 15 mL Falcon tubes for ultrasound induction. Non-induced control cells were also trypsinized and collected. After treatment, cells were reseeded into plates and cultured for an additional 24 h. Total RNA was then extracted using a Quick-RNA Miniprep Kit (Zymo Research, cat. no. R1054) according to the manufacturer's instructions. RNA concentrations were measured using a NanoDrop 2000 spectrophotometer (Thermo Fisher), and samples were diluted to 100 ng/μL for cDNA synthesis. A High-Capacity cDNA Reverse Transcription Kit (Applied Biosystems, cat. no. 4 368 814) was used to generate cDNA following the manufacturer's protocol. The resulting cDNA was diluted five-fold prior to qPCR. SYBR Green Supermix (Bio-Rad, cat. no. 1 725 271) was mixed with the diluted cDNA, and qPCR was performed on a QuantStudio 3 system (Thermo Fisher). GAPDH was used as the housekeeping gene for normalization. Primer sequences used for qPCR are listed in Supplementary Table S2.

**Microencapsulation and implantation of monoclonal cells**. We employed clinically validated, FDA-licensed alginate-based encapsulation technology to protect genetically engineered human monoclonal cells from host immune responses while allowing the diffusion of essential nutrients and therapeutic proteins such as insulin. The alginate microencapsulation process followed standard manufacturing protocols. Briefly, we firstly harvested the stably transfected cells before rinsing with MOPS buffer (10 mM MOPS, pH 7.2, 150 mM NaCl). A total of 50 million cells were suspended in 10 mL of a 1.6% (w/v) sodium alginate solution (Na-alginate, cat. no. 11 061 528, Buechi Labortechnik AG, Switzerland) in a 20 mL syringe, and injected into the encapsulation system (Inotech Encapsulator IE-50R, EncapBiosystems Inc.). During the encapsulation process, 200 mL of 0.05% (w/v) poly-L-lysine (PLL) solution (PLL 2000, cat. no. 25988–63-0, Alamanda Polymers Inc., USA) was used to create coherent alginate-PLL-alginate microcapsules with an average diameter of 400 μm. The encapsulator parameters were as follows: 200 μm nozzle, vibration frequency of 1000 Hz, flow rate of 20 mL/min for the syringe, and 1200 V for bead dispersion. After encapsulation, the cells were cultured in standard DMEM medium for one to three days. The medium was then replaced with serum-free DMEM, and 1.0 mL of the encapsulated alginate beads, each containing 5 × 10^6^ cells, was intraperitoneally implanted on the back of mice using a standard 3 mL syringe (cat. no. 9 400 038, Becton Dickinson) fitted with a 0.7 × 30 mm needle (cat. no. 30382903009009, Becton Dickinson).

**Ultrasonic induction in mice**. Eight-week-old wild-type or T1D male C57BL/6J mice (Janvier Labs) were used in this study. Mice implanted with engineered cells were anesthetized with isoflurane before undergoing ultrasonic induction. Ultrasound gel (cat. no. G00664, Tinovamed GmbH, Switzerland) was applied to the implantation site, and the ultrasound probe was placed over the area to perform the induction. The mice in the treatment group received ultrasonic induction (1.5 W/cm^2^, ∼45 kHz, 40 s) once daily throughout the treatment period. The parameters used for the *in vivo* experiments were consistent with those optimized *in vitro*. Specifically, a sonicator system (cat. no. UCD-600, Diagenode, Belgium) operating at a frequency range of 30–60 kHz and an output power range of 100–350 W (1.25–4.5 W/cm²) was utilized for ultrasonic induction. The sonication program was set to cycles of 5 s on and 5 s off, with a total induction time of 5 s per cycle. Serum samples were collected from the tail vein 24 h post-sonication or at predefined time points to quantify reporter expression or profile therapeutic proteins. **Streptozotocin-induced T1D mouse model**. To induce the T1D mouse model, 8-week-old male wild-type C57BL/6J mice (Janvier Labs) were intraperitoneally injected with freshly prepared streptozotocin (STZ, cat. no. S0130, Sigma-Aldrich; 80 mg/kg in 100 μL sodium citrate buffer, pH 4.3) following an 8-hour fast. This procedure was repeated once daily for four consecutive days. One week after the final injection, the onset of persistent fasting hyperglycemia, a hallmark of T1D, was confirmed by measuring blood glucose levels after an 8-h fast.

**Glucose tolerance test (GTT**). GTT was conducted two to three days after treatment by intraperitoneally administering 1.5 g/kg D-glucose. Blood glucose levels were measured immediately and at regular intervals over a 2-h period.

**Blood glucose profiling**. Blood glucose levels were measured using clinically licensed ContourNext test strips and the ContourNext ONE reader (Ascensia Diabetes Care).

**Animal blood sampling**. Blood samples were drawn from the tail or saphenous veins using 20 μL glass micro-hematocrit capillaries (Avantor® VWR, cat. no. 521–9100) and transferred into BD Microtainer® serum separator tubes (cat. no. BDAM365968, Becton Dickinson). The samples were then centrifuged at 8000 × g for 2 min, and the serum was either immediately analyzed or stored at −80°C within 1 h of collection.

**Insulin quantification assay**. Insulin levels in both blood serum and cell culture supernatants were quantified by an ELISA kit (cat. no. 10–1247-01, Mercordia).

**Animal experiment license**. All animal experiments fully adhered to French or Chinese animal welfare legislation. The experiments were approved by the French Republic (project no. DR2018-40v5 and APAFIS no. 16 753), and conducted by Jinbo Huang, Shuai Xue, and Ghislaine Charpin-El Hamri (no. 69 266 309) at the University of Lyon, Institut Universitaire de Technologie (IUT, F69622 Villeurbanne, France), or performed by Shuai Xue according to the protocol (Protocol ID: AP#24–088-XMQ) approved by the Institutional Animal Care and Use Committee (IACUC) of Westlake University and in accordance with the Animal Care Guidelines of the Ministry of Science and Technology of the People's Republic of China.

**Statistics**. Statistical significance was determined using a two-tailed, unpaired Student's t-test, and one-way or two-way analysis of variance (ANOVA). For analyses involving multiple comparisons, we utilized GraphPad Prism 8 (v 9.2.0, GraphPad Software Inc.) and Microsoft Excel (v 16.51, Microsoft®). Unless otherwise noted, all experiments were conducted with at least two independent biological replicates, and consistent results were observed across the replicates. The number of biological replicates (n), the statistical methods employed, and the significance of differences are detailed in the figures and corresponding legends.

## Results

### Design and validation of a sonogenetic switch in mammalian cells

**Figure 1.**
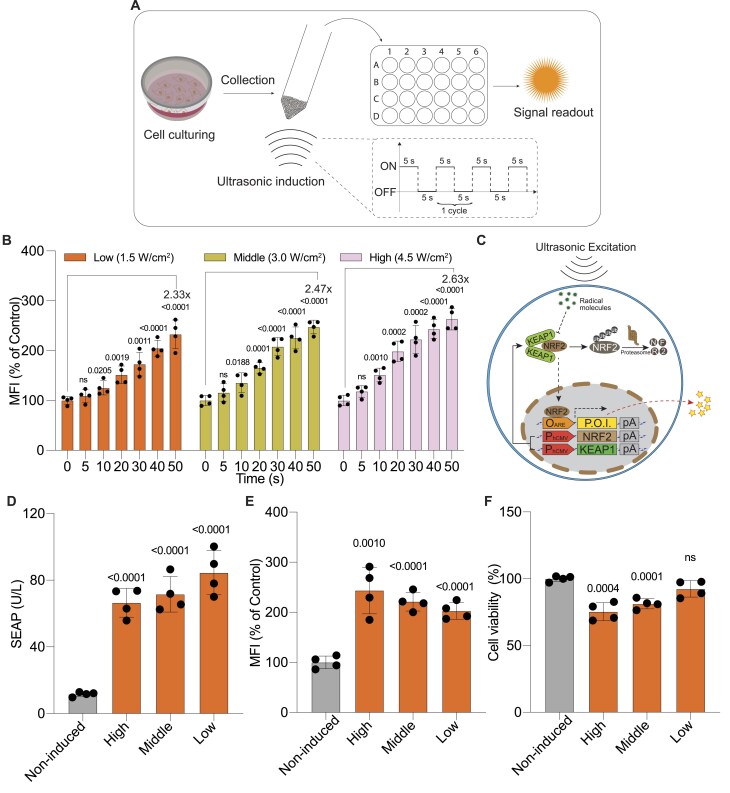
Ultrasound-induced transgene switch design in mammalian cells. (**A**) Experimental setup for ultrasound induction. Cells were cultured in dishes or 24-well plates overnight, then collected into 15 ml Falcon tubes and placed in the sonication water bath for ultrasound induction. After induction, cells were reseeded in 24-well plates. The ON-OFF induction cycle is shown in the dashed box, where induction time represents the total ON duration. (**B**) Quantification of ROS levels in wild-type HEK-293T cells 1 h post-sonication at varying power intensities (high (4.5 W/cm^2^), medium (3.0 W/cm^2^), and low (1.5 W/cm^2^)) for the indicated time periods. (**C**) Schematic illustration of the ultrasound-activated transgene switch based on a hypersensitive ROS sensing system involving engineered NRF2/KEAP1/ARE components (plasmids: pJH1003, pJH1004 and pJH1005). Following the setup in (**A**), engineered cells were cultured in dishes or 24-well plates followed by transfection. Post-transfection, cells containing the ROS-sensing system were collected in 15 ml Falcon tubes for sonication. Under non-induced conditions, constitutively expressed KEAP1 and NRF2 form a complex, leading to continuous NRF2 degradation by the 26S proteasome (solid arrows). Upon ultrasound stimulation, ROS generation is triggered, disrupting the KEAP1–NRF2 interaction. Released NRF2 translocates to the nucleus, where it binds to engineered ARE sites, activating the expression of the protein of interest (P.O.I., represented by stars) (dashed arrows). (**D**) SEAP expression profile 24 h post-sonication at high (4.5 W/cm^2^), medium (3.0 W/cm^2^), and low (1.5 W/cm^2^) ultrasound power levels for 40 s in engineered cells transiently transfected with the ROS sensing system (plasmids: pJH1003, pJH1004, and pJH1005). The samples were collected from the cell culture supernatant. (**E** and **F**) Quantification of ROS levels (**E**) and cell viability (**F**) in engineered cells post-sonication at varying power intensities (high (4.5 W/cm^2^), medium (3.0 W/cm^2^), and low (1.5 W/cm^2^)). The ROS levels were quantified 1 h after sonication (**E**), while the cell viability was evaluated 24 h post-sonication (**F**). “Non-induced” refers to cells without ultrasound stimulation. Data are presented as mean ± SD; *n* = 4. “ns” indicates not significant (*P* value > 0.05). Statistical significance (*P* values) was calculated between induced and non-induced groups.

To develop an ultrasound-responsive transgenic switch, we first examined whether ultrasonic excitation could induce the production of radical signal molecules within mammalian cells (Fig. 1A). Indeed, upon applying ultrasound stimulation within a certain range of parameters (>1.5 W/cm^2^, >30 kHz, >10 s), we observed a significant increase in intracellular ROS (Fig. 1B), with minimal impact on cell viability at exposures of up to 40 s (Supplementary Fig. S1A). Notably, the ROS levels induced by short-term ultrasound exposure (< 40 s) were comparable to those triggered by low concentrations (<50 μmol/L) of the classical ROS inducer tert-butylhydroquinone (tBHQ) [48, 49] (Supplementary Fig. S1B) without affecting cell viability. This finding led us to investigate the potential of linking ultrasound stimulation to gene expression through the KEAP1/NRF2 ROS-sensing pathway, which includes KEAP1, functioning as a ROS sensor and regulator, and NRF2, a transcriptional activator that responds to ROS signals by inducing expression of gene of interest via engineered ARE promoters (Fig. 1C).

For better operability in this setup, we seeded HEK-293T cells in culture dishes and co-transfected them with plasmids (pJH1003, pJH1004, and pJH1005) containing the ROS-sensitive gene expression system. Prior to ultrasonic stimulation, cells were collected into tubes, and after ultrasound exposure, the cells were returned to culture plates for continued growth and assessment of reporter gene expression (Fig. 1C). We detected a remarkable increase in SEAP (human secreted alkaline phosphatase) reporter expression following ultrasound stimulation at different power settings for 40 s (Fig. 1D). ROS quantification assay further confirmed that ultrasound exposure activated the ROS-sensing system, as indicated by elevated levels of radicals within the cells (Fig. 1E). However, cell viability assay indicated that high-intensity ultrasound (e.g. >3.0 W/cm^2^) resulted in increased cytotoxicity (Fig. 1F). Based on these observations, we employed low-intensity ultrasound (1.5 W/cm^2^, ∼45 kHz) in subsequent experiments to minimize cytotoxicity.

### Characterization of the SUPER system in transiently transfected cells

**Figure 2.**
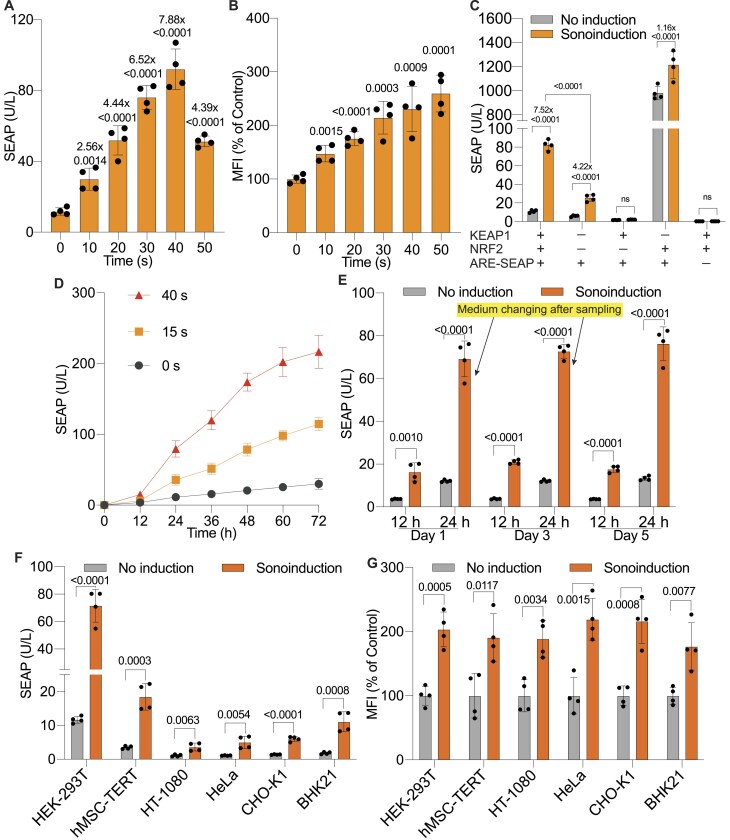
*In vitro* characterization and validation of the sonogenetic switch. (**A**) SEAP levels in the cell culture supernatant of engineered HEK-293T cells co-transfected with the ROS-sensing system (plasmids: pJH1003, pJH1004, and pJH1005) after 24 h of ultrasound induction. Low-intensity ultrasonic stimulation was applied for the indicated durations. Induction folds relative to the non-induced control are shown on the top of each column. (**B**) Quantification of ROS levels in engineered HEK-293T cells containing the ROS-sensing system 1 h post-ultrasound stimulation for the specified time periods. (**C**) SEAP produced by cells transfected with ARE reporter (P_ARE_-SEAP, pJH1005) alone and/or in combination with other components (KEAP1, pJH1004; NRF2, pJH1003). Engineered cells were induced using low-intensity ultrasound (1.5 W/cm^2^, ∼45 kHz) for 40 s. (**D**) Kinetics of SEAP reporter expression in the culture supernatant of engineered HEK-293T cells transfected with the ROS-sensing system over three days following ultrasonic induction (1.5 W/cm^2^, ∼45 kHz). A single ultrasound induction was applied at the 0-hour time point for the specified duration. (**E**) Reversibility of the ultrasound-induced system in transiently transfected mammalian cells. Engineered HEK-293T cells were alternately cultured in 24-h cycles with ultrasonic treatment (Sonoinduction; 1.5 W/cm^2^, ∼45 kHz, 40 s) or without ultrasound (No induction). The cell culture medium was replaced, and cell density was readjusted at the end of days 1 and 3. Low-intensity ultrasound (1.5 W/cm^2^, ∼45 kHz) induction for 40 s was then reapplied after 24 h of stimulus-free culturing. SEAP production was measured every 12 h in the culture supernatant after each ultrasound induction. F. Versatility of the sonogenetic system. SEAP levels were measured in the supernatant of different mammalian cell lines transiently transfected with the sonogenetic system and subjected to low-intensity ultrasound for 40 s. (**G**) Quantification of ROS levels in the indicated mammalian cell lines exposed to low-intensity ultrasound for 30 s. Data are presented as mean ± SD; *n* = 4. The statistical significance (*P* values) of differences was calculated between induced and non-induced groups, or as indicated.

We next analyzed the time course of transgene expression in response to low-intensity ultrasound stimulation (1.5 W/cm^2^, ∼45 kHz). This induced a time-dependent increase in reporter gene expression, peaking at an exposure time of 40 s (Fig. 2A). Longer stimulation durations resulted in more elevated ROS levels (Fig. 2B), which were associated with reduced cell viability (Supplementary Fig. S2A). Therefore, we chose ultrasonic stimulation for 40 sec for the following experiment. To evaluate the necessity of each component in the ROS-sensing system, we transfected mammalian cells with the ARE-SEAP reporter plasmid (pJH1005) alone or in combination with other components. Ultrasound induction (1.5 W/cm^2^, ∼45 kHz, 40 s) of HEK-293T cells transfected exclusively with the ARE-SEAP reporter resulted in significantly reduced SEAP expression levels and a lower induction fold compared to cells co-expressing KEAP1 and NRF2 (Fig. 2C). These findings suggest that endogenous levels of KEAP1 and NRF2 are insufficient to fully support the system's optimal functionality, while their ectopic co-expression markedly enhances the sensitivity of the engineered cells to ROS production triggered by ultrasound stimulation. To investigate the kinetics of SEAP production, we monitored the accumulation of SEAP over a 3-day period following ultrasonic stimulation at different induction durations (Fig. 2D). The sonogenetic switch provided robust ON/OFF control, showing consistent induction and repression profiles across multiple ON-to-OFF and OFF-to-ON cycles with fresh medium changes at 24-h intervals (Fig. 2E).

To evaluate the versatility of the engineered gene switch, we conducted transient transfections in various mammalian cell lines. Although there were differences in SEAP expression level and induction fold across cell types following ultrasound exposure, the gene switch remained functional in all tested lines (Fig. 2F and Supplementary Fig. S2B). This included human mesenchymal stem cell-derived hMSC-TERT cells, indicating a broad cellular compatibility and potential for diverse applications. Furthermore, ROS quantification confirmed consistent ROS generation efficiency across these cell lines under ultrasonic stimulation, supporting the reliability of the system (Fig. 2G).

### Characterization of the SUPER system for therapeutic protein expression in monoclonal cells

**Figure 3.**
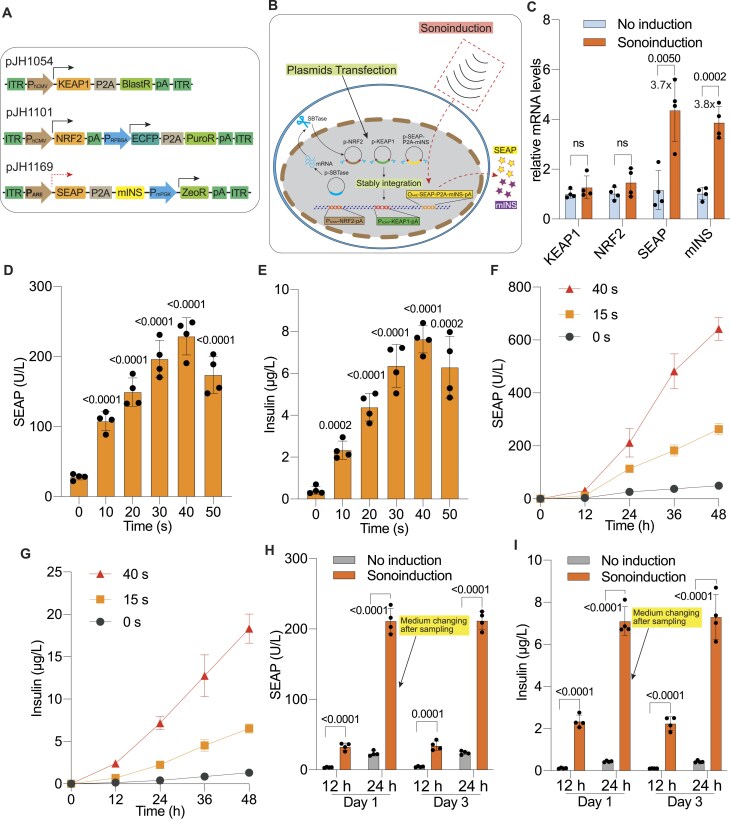
In vitro characterization of the ultrasound-responsive stable cell line. (**A**) Schematic of DNA constructs used to generate the monoclonal cell line. The ROS-sensing system (pJH1054/pJH1101/pJH1169) was stably integrated into the mammalian cell genome using the SB100X Sleeping Beauty transposase, which targets and recognizes the flanking inverted terminal repeat (ITR) elements in the constructs. (**B**) Schematic of stably transfected hMSC-TERT cells designed to express SEAP and mouse insulin (mINS) upon ultrasound induction. Cells were co-transfected with plasmids (pJH1054, pJH1101, pJH1169) containing the ROS-responsive system flanked by Sleeping Beauty (SB) transposase recognition sites, along with a plasmid for constitutive SB transposase expression (pJH42). After antibiotic selection, the ultrasound-responsive system was stably integrated into the genome (solid arrows). Upon ultrasound induction, cells produce SEAP and mINS (dashed arrows). (**C**) Relative mRNA expression levels of KEAP1, NRF2, SEAP, and mINS in engineered stable cells, either non-induced or after ultrasound stimulation (sonoinduction). Cells were harvested 24 h after induction for qPCR analysis. (**D** and **E**) Quantification of SEAP (**D**) and insulin (**E**) levels in the supernatant of monoclonal stable cell cultures following ultrasound stimulation with low-intensity power (1.5 W/cm^2^, ∼45 kHz) for the indicated durations. (**F** and **G**) Kinetics of SEAP (**F**) and insulin (**G**) expression over 2 days in monoclonal cells after ultrasound induction (1.5 W/cm^2^, ∼45 kHz) for the specified time periods. (**H** and **I**) Reversibility assessment of SEAP (**H**) and insulin (**I**) expression in stable cells during alternating ON (Sonoinduction) and OFF (No induction) cycles. The cell culture medium was replaced, and cell density was readjusted at the end of day 1. Low-intensity ultrasound (1.5 W/cm^2^, ∼45 kHz) induction for 40 s was applied to the engineered cells. Supernatant samples were collected every 12 h to measure SEAP (**H**) and insulin (**I**) levels. Data are presented as mean ± SD; *n* = 4. “ns” indicates not significant (*P* value > 0.05). *P* values was calculated between induced and non-induced groups, or as indicated.

Diabetes, a chronic metabolic disorder with a rapidly increasing global prevalence, requires dynamic and precise management. This motivated us to use diabetes as a proof-of-concept model for our ultrasound-responsive gene expression system. To achieve stable expression of therapeutic proteins in response to ultrasound, we tested a monoclonal cell line [21] containing an ROS-sensitive gene cassette integrated into the genome via the Sleeping Beauty transposase system. This cassette drives the expression of both SEAP and mouse insulin (mINS) as outputs (Fig. 3A and B). The selected monoclonal cell line, hereafter referred to as SUPER_INS_, exhibited significantly higher levels of ultrasound-induced SEAP expression compared to transiently transfected cells (Supplementary Fig. S3A), making it a suitable candidate for further characterization.

To evaluate the transcriptional response of SUPER_INS_ cells, we conducted qPCR to measure the relative mRNA levels of key genes (KEAP1, NRF2, SEAP, and mINS) under induced and non-induced conditions. The constitutively expressed genes, KEAP1 and NRF2, showed consistent transcript levels regardless of the treatment, whereas SEAP and mINS transcripts were significantly upregulated in the ultrasound-induced group, showing an increase of 3.7-fold and 3.8-fold, respectively, compared to the non-induced control (Fig. 3C). These findings confirm the effective ultrasound inducibility of the SUPER_INS_ cell line.

Further analysis of the time course of the response demonstrated that the induction patterns of SUPER_INS_ cells were comparable to those observed in transiently engineered cells within a 50-s exposure window. SEAP (Fig. 3D) and insulin (Fig. 3E) expression levels both peaked at 40 s of ultrasonic stimulation (Supplementary Fig. S3B and Supplementary Fig. S3C), which is consistent with the response in the case of transient transfection (Fig. 2A). We also investigated the expression kinetics of SEAP and mINS over a 48-h period following ultrasound stimulation. The expression profiles for both SEAP (Fig. 3F) and mINS (Fig. 3G) were dose- and time-dependent, demonstrating sustained induction post-stimulation. Additionally, the system displayed robust reversibility, as SEAP (Fig. 3H) and mINS (Fig. 3I) expression could be reliably toggled between ON and OFF states at 24-h intervals, confirming the system's stability and controllability.

### Non-invasive ultrasound exposure induces insulin expression in T1D mice

**Figure 4.**
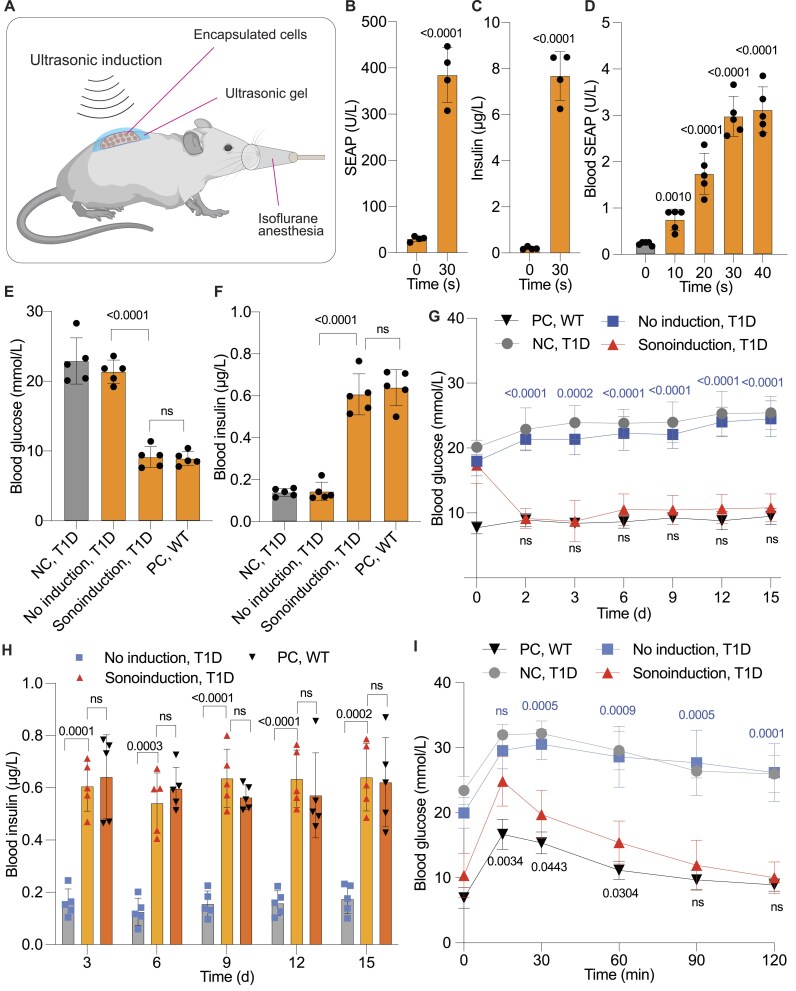
In vivo functionality evaluation and validation of the ultrasound-responsive system. (**A**) Schematic illustration of the procedure for implanting encapsulated engineered monoclonal cells subcutaneously into the dorsal region of mice and performing ultrasonic induction. Ultrasound gel was applied to the implantation site to ensure conductivity and temperature regulation. Mice were anesthetized using an isoflurane system during implantation and ultrasound induction to facilitate operability. (**B** and **C**) SEAP (**B**) and insulin (**C**) expression in stable cells encapsulated in alginate beads. Encapsulated cells were cultured in 24-well plates with DMEM medium supplemented with 10% FBS, and stimulated with ultrasound for 40 s. SEAP (**B**) and insulin (**C**) levels were measured in the supernatant after a 24-h induction. (**D**) Dose-dependent SEAP expression in blood following low-intensity ultrasound induction at the specified time intervals. Ultrasound induction was performed every 12 h post-implantation of engineered stable cells. (**E** and **F**) Measurements of fasting blood glucose (**E**) and insulin levels (**F**) in T1D mice. Fasting glucose and insulin levels were recorded after 8 h of fasting, following two days of ultrasonic treatment. Mice injected with encapsulated monoclonal cells received either no treatment (No induction) or ultrasound treatment (Sonoinduction, low-intensity for 30 seconds). Except for the WT group, all groups consisted of T1D mice. (**G**) Monitoring of fasting glucose levels during the treatment period. Fasting glucose was measured before implantation (day 0) and for 15 days after implantation in the indicated groups. The data for day 2 are replotted from Fig. 4E. (**H**) Quantification of blood insulin levels during the treatment period. Insulin levels were measured in WT mice without implantation, T1D mice with implants subjected to ultrasound induction, and T1D mice with implants but without induction. In (**G**) and (**H**), the mice in the treatment group received ultrasonic induction (1.5 W/cm^2^, ∼45 kHz, 40 s) once daily throughout the entire treatment period. (**I**) Intraperitoneal glucose tolerance test (ipGTT). After 3 days of ultrasonic treatment, D-glucose was administered intraperitoneally (1.5 g/kg) after an 8-h fast, and glucose tolerance was evaluated. Different colors in statistical notations correspond to comparisons between data points of the same color group and the sonoinduced group. In panels (**E**–**I**), WT and T1D mice without implants or treatment served as controls. NC: negative control, PC: positive control. Data are presented as mean ± SD; *n* = 5. “ns” indicates non-significance (*P* > 0.05). *P* values indicate the significance of differences between induced and non-induced groups or as specified.

For *in vivo* proof-of-concept studies, we encapsulated the monoclonal SUPER_INS_ cells within alginate beads, which provides two major benefits: the alginate shields the engineered cells from the host's immune response and allows the free diffusion of nutrients and therapeutic proteins [50]. The alginate beads were then implanted subcutaneously (s.c.) on the dorsal side of mice to enable non-invasive ultrasound induction. To protect the surrounding tissue and implanted cells from potential heat damage due to ultrasound, the implantation site was evenly coated with ultrasound gel, which also improved ultrasound penetrability for efficient induction (Fig. 4A). Throughout the implantation and induction procedures, mice were kept under anesthesia to ensure stability and accuracy of the experiments (Fig. 4A).

Before proceeding with the *in vivo* experiments, we verified the expression performance of SEAP (Fig. 4B) and mINS (Fig. 4C) by the engineered cells microencapsulated in alginate beads. Both proteins were successfully induced and detected in the culture medium (Fig. 4B and C), confirming the permeability of the alginate matrix to both the reporter and therapeutic proteins. To evaluate the *in vivo* capability of the SUPER system, the encapsulated cells were subcutaneously implanted into wild-type (WT) mice and exposed to ultrasound treatments of varying duration (Fig. 4D). Blood analysis demonstrated a dose-dependent increase in SEAP expression in response to ultrasound induction, confirming that transgene expression in the implanted cells can be precisely regulated via ultrasound (Fig. 4D). Given that 40 s of ultrasound afforded the highest induction of SEAP in the implanted mice, we selected a fixed duration of 40 s for induction in subsequent experiments.

Next, to examine the regulation of therapeutic protein expression, we implanted the alginate-encapsulated SUPER_INS_ cells into T1D mice. Ultrasound treatment significantly reduced fasting blood glucose levels in the T1D mice (Sonoinduction, T1D) to levels comparable to those in WT mice (Positive Control, PC, WT) (Fig. 4E). In contrast, blood glucose levels in T1D mice without ultrasonic induction (No Induction, T1D) remained elevated and similar to those in the negative control group (NC, T1D) that had neither implant nor induction (Fig. 4E). Blood insulin levels in the ultrasound-treated group were restored to WT levels, while insulin levels remained low in the non-induced groups (Fig. 4F).

Blood glucose monitoring over a 15-day period showed that mice treated with ultrasound once per day maintained normoglycemia throughout the entire treatment period, with blood glucose values comparable to those in the WT group. In contrast, non-induced mice maintained significantly higher glycemic levels over the whole treatment period (Fig. 4G). Blood insulin profiles further supported these findings, showing significantly elevated insulin levels (>0.5 μg/L) in ultrasound-induced mice compared to non-induced implanted mice, which displayed much lower insulin levels (<0.2 μg/L) (Fig. 4H). An intraperitoneal glucose tolerance test (ipGTT) conducted three days post-implantation demonstrated that ultrasound-induced insulin production not only restored glucose homeostasis, but also attenuated postprandial blood glucose spikes compared to control groups without implants or those with non-induced implants (Fig. 4I). Collectively, these findings indicate that the SUPER system is a promising approach to achieve precise control of therapeutic transgene expression with a non-invasive inducer.

## Discussion

In this study, we developed and validated an ultrasound-activated synthetic gene circuit capable of precise and reversible control of transgene expression. By integrating a ROS-responsive genetic switch, consisting of ROS sensors KEAP1/NRF2 and an ARE-responsive output, into mammalian cells, we demonstrated that ultrasound stimulation can reliably trigger the production of both reporter (SEAP) and therapeutic (mINS) proteins. The ultrasound parameters could be finely tuned to achieve optimal gene expression without impacting on cell viability. Notably, ultrasound stimulation of subcutaneously implanted, alginate-encapsulated cells expressing this gene regulation platform restored normoglycemia in a T1D mouse model.

Compared with traditional gene regulation systems that rely on small molecules such as doxycycline, ABA, and rapamycin [7–9, 12], our ultrasound-based platform offers precise, localized, and immediate control over gene expression. Its parameter flexibility allows for fine-tuning of expression levels, overcoming the limitations of slower and less dynamic small-molecule inducers, which are often delivered systemically, raising concerns around off-target effects and the need for repeated dosing to maintain efficacy [2, 10–12]. We believe these advantages make our system an excellent candidate for precision medicine applications, where adaptable, localized gene control is critical. Our approach also provides significant advantages over other physical gene regulation methods, including earlier ultrasound-based systems. For example, optogenetics faces challenges due to the limited tissue penetration of light and the need for invasive optical hardware [25, 26], while electrogenetic systems depend on electronic interfaces that can cause cytotoxicity and immunogenicity [15, 16, 21, 27]. Further, previous ultrasound-based systems have generally leveraged either ultrasound's mechanical force or heating effect [15, 31, 34, 35, 38], which may cause tissue damage, or employed nanoparticles, raising biocompatibility concerns [39, 40]. In contrast, our low-intensity ultrasound approach enables deep tissue penetration without compromising cellular function or requiring hardware implantation, circumventing these issues. Specifically, our system enables safe, tunable, and reversible modulation of gene expression via low-level ROS generation using a brief 40-second exposure to low-intensity ultrasound (1.5 W/cm²) to control gene expression. Furthermore, we confirmed robust reversibility in repeated on-off cycles of gene expression, as well as compatibility with various cell types, including human mesenchymal stem cell-derived hMSC-TERT cells.

We believe this approach enhances the practicality and safety of ultrasound for therapeutic use by providing a non-invasive, mediator-free, and reversible platform with potential for a broad range of clinical applications in regenerative medicine, chronic disease management, and personalized therapies. Indeed, the successful application of our developed SUPER system to normalize hyperglycemia in a type 1 diabetes mouse model underscores the potential of ultrasound-induced gene expression for therapeutic applications in precision medicine. Integrating this system with advanced gene-editing technologies, such as CRISPR-Cas9, could also enable highly specific, minimally invasive genome modifications in the future.

## Supplementary Material

gkaf191_Supplemental_File

## Data Availability

The authors declare that all the data supporting the findings of this study are available within the paper and its supplementary materials. Original plasmids are available upon reasonable request. All vector information is provided in Supplementary Table S1.

## Notes

Present address: Westlake Laboratory of Life Sciences and Biomedicine, Hangzhou, Zhejiang, China
