# Supplementary material for: A mediator-free sonogenetic switch for therapeutic protein expression in mammalian cells
Source: Nucleic Acids Res. 2025 Mar 20;53(6):gkaf191. doi: 10.1093/nar/gkaf191 (PMC11925730; doi:10.1093/nar/gkaf191)
Supplement: gkaf191_Supplemental_File [file gkaf191_supplemental_file.docx]

**Supplementary Information**

**A mediator-free sonogenetic switch for therapeutic protein expression in mammalian cells**

Jinbo Huang^1^, Shuai Xue^1,2^, Ana Palma Teixeira^1^, Martin Fussenegger^1,3,*^

^1^Department of Biosystems Science and Engineering, ETH Zurich, Klingelbergstrasse 48, CH-4056 Basel, Switzerland.

^2^Present address: Westlake Laboratory of Life Sciences and Biomedicine, Hangzhou, Zhejiang, China.

^3^Faculty of Science, University of Basel, Klingelbergstrasse 48, CH-4056 Basel, Switzerland.

^*^Corresponding author. E-mail: fussenegger@bsse.ethz.ch

This file includes:

- Supplementary Figures: 1-3
- Supplementary Tables: 1-2

**Supplementary Figure S1. Effect of ultrasound and chemical stimulation on mammalian cell viability. A**. Cell viability was assessed using a resazurin assay across all experimental groups subjected to ultrasound exposure at the specified time intervals. **B.** Quantitative analyses of reactive oxygen species (ROS) levels in wild-type HEK-293T cells 1 h post-incubation with tBHQ at various concentrations. ROS induction is shown as the mean fluorescence intensity (MFI) with respect to the untreated group taken as 100%. **C**. Viability analysis for wild-type HEK-293T cells treated with tBHQ at various concentrations. After 24 h incubation, cell viability was assessed using the resazurin assay. Results are shown as mean ± SD (n = 4) for each group. Statistical analysis was conducted to compare the viability of ultrasound-treated groups with that of the non-stimulated control group. The *P* value is shown for values < 0.05 compared to the non-induced control group. “ns” denotes no significant difference (*P* > 0.05) from the control group in all cases, indicating that ultrasound exposure under these conditions did not adversely impact cell viability.

**Supplementary Figure S2. Effect of Ultrasound Stimulation on the Viability of Mammalian Cells Transfected with ROS-Sensing Systems. A-B.** Mammalian cells were co-transfected overnight with ROS-responsive plasmids (pJH1003/pJH1004/pJH1005) prior to ultrasound exposure. Cells were subsequently stimulated with low-intensity ultrasound for various durations (**A**) or a fixed 40-second duration (**B**). Cell viability was assessed using the resazurin assay, with results shown as mean ± SD (n = 4) for each condition. Statistical analysis was conducted to compare each ultrasound-treated group with the non-stimulated control group. The *P* value is shown for values < 0.05. “ns” denotes no significant difference (*P* > 0.05), indicating no adverse effect on cell viability under these conditions for exposures up to 40 seconds.

**Supplementary Figure S3. Effects of ultrasound stimulation on monoclonal cells harboring the ROS-sensing system. A**. Preliminary testing of the response of the stable cell line to ultrasound exposure, with 30 seconds of induction at the specified intensity. **B-C.** Quantitative analyses of reactive oxygen species (ROS) levels (**B**) and cell viability (**C**) in engineered monoclonal cells following ultrasound induction over various time points. Data are presented as mean ± SD (n = 4). The *P* value is shown for values < 0.05 compared to the non-induced control group. “ns” indicates no significant difference (*P* > 0.05) compared to the control. MFI: mean fluorescence intensity.

**Supplementary Table S1.** Plasmids used and designed in this study.

| **Plasmid** | **Description** | **Reference** |
| --- | --- | --- |
| BB6-BlastR | SB100X-specific transposon containing a constitutive BlastR and iRFP expression unit.  (ITR-MCS:P_hCMV_-BlastR-P2A-iRFP-pA-ITR). | Huang et al. (1) |
| BB6-PuroR | ﻿SB100X-specific transposon containing a constitutive ECFP and PuroR expression unit.  (ITR-MCS:P_RPBSA_-ECFP-P2A-PuroR-pA-ITR). | Huang et al. (1) |
| BB6-ZeoR | SB100X-specific transposon containing a constitutive ZeoR and mRuby expression unit.  (ITR-MCS-pA: P_hCMV_-ZeoR-P2A-mRuby-pA-ITR). | Huang et al. (1) |
| Mkp37 | Constitutive mammalian TetR-Elk1 fusion protein expression vector (P_hCMV_-TetR-Elk1-pA). | Keeley et al. (2) |
| H107 | Constitutive mammalian expression vector.  (P_hCMV_-eGFP-3FLAG:P_mPGK_-ZeoR-pA). | ObiO, Shanghai |
| pcDNA3.1(+) | Constitutive mammalian expression vector ﻿containing a NeoR resistance gene. (P_hCMV_-MCS-pA). | Thermo Fisher Scientific, CA |
| pCK53 | CRE-driven SEAP expression vector. (P_CRE_-SEAP-pA). | Kemmer et al.(3) |
| pdCas9-VPR | Constitutive dCas9-VPR expression vector. (P_hCMV_- dCas9-VPR-pA) (Addgene no. 63798). | Chavez et al.(4) |
| phIR | Constitutive mammalian expression vector containing human insulin receptor (hIR) gene.  (P_hCMV_-hIR-pA). | Ray et al. (5) |
| pMF111 | Mammalian reporter plasmid for TetR-Elk1-induced SEAP expression. (﻿P_TRE_-SEAP-pA). | Fussenegger et al.(6) |
| pXS101 | SB100X-specific transposon containing a constitutive mammalian promotor-driven CREB1-TetR stable expression vector in mammalian cells. (ITR-P_hCMV_-CREB1-TetR-2A-mCherry-2A-PuroR-pA-ITR). | Xue et al.(7) |
| pJH3 | Constitutive mammalian SEAP expression vector. (P_hCMV_-SEAP-pA). | Huang et al. (1) |
| pJH5 | Constitutive mammalian mCherry expression vector. (P_hCMV_-mCherry-pA). | Huang et al. (1) |
| pJH42 | Constitutive SB100X expression vector. (P_hCMV_-SB100X-pA). | Huang et al. (1) |
| pJH43 | Constitutive mammalian expression vector ﻿containing a NeoR resistance gene. (P_SV40_-MCS-pA). | Huang et al. (8) |
| pJH44 | Constitutive mammalian expression vector ﻿containing a NeoR resistance gene. (P_EF-1α_-MCS-pA). | Huang et al. (8) |
| pJH45 | Constitutive mammalian expression vector ﻿containing a NeoR resistance gene. (P_mPGK_-MCS-pA). | Huang et al. (8) |
| pJH1003 | Constitutive NRF2 expression vector (P_hCMV_-NRF2-pA). | Huang et al. (1) |
| pJH1004 | Constitutive KEAP1 expression vector (P_hCMV_-KEAP1-pA). | Huang et al. (1) |
| pJH1005 | ARE-driven SEAP expression vector (P_ARE_-SEAP-pA). | Huang et al. (1) |
| pJH1006 | Two tandem ARE-driven SEAP expression vector (P_ARE2_-SEAP-pA). | Huang et al. (1) |
| pJH1009 | Three tandem ARE-driven SEAP expression vector (P_ARE3_-SEAP-pA). | Huang et al. (1) |
| pJH1010 | Four tandem ARE-driven SEAP expression vector (P_ARE4_-SEAP-pA). | Huang et al. (1) |
| pJH1053 | SB100X-specific transposon containing a constitutive NRF2 and ZeoR expression unit  (ITR-P_hCMV_-NRF2-P2A-ZeoR-pA-ITR). | Huang et al. (1) |
| pJH1054 | SB100X-specific transposon containing a constitutive KEAP1 and BlastR expression unit  (ITR-P_hCMV_-KEAP1-P2A-BlastR-pA-ITR). | Huang et al. (1) |
| pJH1101 | SB100X-specific transposon containing a constitutive NRF2 expression unit and a constitutive ECFP and PuroR expression unit  (ITR-P_hCMV_-NRF2-pA: P_RPBSA_-ECFP-P2A-PuroR-pA-ITR). | Huang et al. (1) |
| pJH1102 | SB100X-specific transposon containing a constitutive KEAP1 expression unit and a constitutive BlastR and iRFP expression unit  (ITR-P_hCMV_-KEAP1-pA:P_hCMV_-BlastR-P2A-iRFP-pA-ITR). | Huang et al. (1) |
| pJH1157 | Four tandem ARE-driven SEAP and mouse insulin expression vector (P_ARE4_-SEAP-P2A-mINS-pA) | Huang et al. (1) |
| pJH1159 | SB100X-specific transposon containing a four tandem ARE-driven SEAP and insulin expression unit and a constitutive ECFP and PuroR expression unit  (ITR-P_ARE4_-SEAP-P2A-mINS-pA:P_RPBSA_-ECFP-P2A-PuroR-pA-ITR). | Huang et al. (1) |
| pJH1169 | SB100X-specific transposon containing a four tandem ARE-driven SEAP and insulin expression unit and a constitutive ZeoR expression unit  (ITR-P_ARE4_-SEAP-P2A-mINS:P_mPGK_-ZeoR-pA-ITR). | Huang et al. (1) |

**Abbreviations:**

**ARE**: antioxidant response element; **BlastR**, gene conferring blasticidin resistance; **cDNA**, complementary DNA; **CMV**, cytomegalovirus; **CREB1**, CAMP-responsive element binding protein 1; **dCas9**, nuclease-deactivated Cas9 endonuclease; **ECFP**, enhanced cyan fluorescent protein; **EGFP**, enhanced green fluorescent protein; **Elk1**, ETS-like-1 transcription factor; **FC**, fragment crystallizable region of antibody; **FLAG**, FLAG octapeptide tag; **iRFP**, near-infrared fluorescent protein; **ITR**, inverted terminal repeats of SB100X; **MCS**, multiple cloning site; **mINS**, modified insulin variant for optimal expression in HEK-293 cells; **mPGK**: a mouse constitutive promoter; **mRuby**: a bright monomeric red fluorescent protein; **NRF2**: nuclear factor erythroid 2 p45-related factor 2; **O_tetR_**, TetR-specific operator; **P2A**, picornavirus-derived ribosome skipping sequence optimized for bicistronic expression in mammalian cells; **pA**, polyadenylation signal; **P_EF-1a_**, human elongation factor-1 alpha promoter; **P_hCMV_**, human cytomegalovirus immediate early promoter; **P_hCMVmin_**, minimal version of P_hCMV_; **P_RPBSA_**: a constitutive synthetic mammalian promotor; **P_SV40_**, simian virus 40 promoter; **P_TRE_**, O_TetR_-P_hCMVmin_; **PuroR**, gene conferring puromycin resistance; **SB100X**, optimized Sleeping Beauty transposase; **SEAP**, human placental secreted alkaline phosphatase; **T7**, T7 promoter; **TetR**, *Escherichia coli* Tn10-derived tetracycline-dependent repressor of the tetracycline resistance gene; **VP16**, herpes simplex virus protein 16 transactivation domain; **VP64**, a transcriptional activator composed of four tandem copies of VP16; **VPR**, tripartite transcriptional activator consisting of VP64, P65 and Rta; **ZeoR**, gene conferring zeocin resistance; *, stop codon.

**Supplementary Table S2.** Primers used for qPCR analysis.

| Gene | Forward primer | Reverse primer |
| --- | --- | --- |
| *KEAP1* | 5’- TTGGCATCATGAACGAGCTGG-3’ | 5’-TGAAGACAGGGCTGGATGAG-3’ |
| *NRF2* | 5’-TGAGCCCAGTATCAGCAACA-3’ | 5’- CTGTGCTTTCAGGGTGGTTT-3’ |
| *SEAP* | 5’-CCAAATGGGCCAGATCGAAA-3’ | 5’-TGTGCCATAGTGCAGGATCA-3’ |
| *Insulin* | 5’-GGGATCTTCAGACCTTGGCA-3’ | 5’-TGCAGTAGTTCTCCAGTTGGT-3’ |
| *GAPDH* (human house-keeping gene) | 5’-GTCTCCTCTGACTTCAACAGCG-3’ | 5’-ACCACCCTGTTGCTGTAGCCAA-3’ |

**References:**

1. Huang, J., Xue, S., Buchmann, P., Teixeira, A.P. and Fussenegger, M. (2023) An electrogenetic interface to program mammalian gene expression by direct current. *Nature Metabolism*, 1-13.

2. Keeley, M.B., Busch, J., Singh, R. and Abel, T. (2005) TetR hybrid transcription factors report cell signaling and are inhibited by doxycycline. *BioTechniques*, **39**, 529-536.

3. Kemmer, C., Gitzinger, M., Daoud-El Baba, M., Djonov, V., Stelling, J. and Fussenegger, M. (2010) Self-sufficient control of urate homeostasis in mice by a synthetic circuit. *Nature Biotechnology*, **28**, 355-360.

4. Chavez, A., Scheiman, J., Vora, S., Pruitt, B.W., Tuttle, M., Iyer, E.P., Lin, S., Kiani, S., Guzman, C.D. and Wiegand, D.J. (2015) Highly efficient Cas9-mediated transcriptional programming. *Nature Methods*, **12**, 326-328.

5. Guha Ray, P., Maity, D., Huang, J., Zulewski, H. and Fussenegger, M. (2023) A versatile bioelectronic interface programmed for hormone sensing. *Nature Communications*, **14**, 3151.

6. Fussenegger, M., Bailey, J.E. and Varner, J. (2000) A mathematical model of caspase function in apoptosis. *Nature Biotechnology*, **18**, 768-774.

7. Xue, S., Yin, J., Shao, J., Yu, Y., Yang, L., Wang, Y., Xie, M., Fussenegger, M. and Ye, H. (2017) A synthetic-biology-inspired therapeutic strategy for targeting and treating hepatogenous diabetes. *Molecular Therapy*, **25**, 443-455.

8. Huang, J., Xue, S., Xie, Y.Q., Teixeira, A.P. and Fussenegger, M. (2024) Ultrashort‐Peptide‐Responsive Gene Switches for Regulation of Therapeutic Protein Expression in Mammalian Cells. *Advanced Science*, 2309411.
